# Nonfunctional adrenocortical carcinoma initially presenting as retroperitoneal hemorrhage

**DOI:** 10.1186/s12893-015-0031-3

**Published:** 2015-04-18

**Authors:** Shinichiro Kashiwagi, Ryosuke Amano, Naoyoshi Onoda, Satoru Noda, Keiichiro Hirata, Yuka Asano, Kento Kurata, Koutaro Miura, Sadaaki Yamazoe, Kenjiro Kimura, Masahiko Ohsawa, Seiichi Kitagawa, Kosei Hirakawa

**Affiliations:** Department of Surgical Oncology, Osaka City University Graduate School of Medicine, 1-4-3 Asahi-machi, Abeno-ku Osaka, 545-8585 Japan; Department of Diagnostic Pathology, Osaka City University Graduate School of Medicine, 1-4-3 Asahi-machi, Abeno-ku Osaka, 545-8585 Japan; Department of Physiology, Osaka City University Graduate School of Medicine, 1-4-3 Asahi-machi, Abeno-ku Osaka, 545-8585 Japan

**Keywords:** Adrenocortical carcinoma, Retroperitoneal hemorrhage, Mitotane, Interventional radiology, Surgery

## Abstract

**Background:**

Acute adrenal hemorrhage is an uncommon entity. Although trauma is the most common cause of adrenal hemorrhage, non-traumatic etiologies have also been reported. We report an unusual case of a spontaneously ruptured adrenocortical carcinoma that initially presented as a critical massive retroperitoneal hemorrhage. The case was treated successfully using a combination of emergency interventional radiology and elective surgery.

**Case presentation:**

A 47-year-old woman was transported to our hospital because of the sudden onset of severe pain in her left lower back. The shadow of a tumor-like soft mass accompanied by bleeding was observed in the upper pole of the left kidney, together with vascular leakage from the middle suprarenal artery on computed tomography. Transcatheter embolization of the left middle adrenal artery was administered based on a diagnosis of acute adrenal hemorrhage. Further observation indicated that the bleeding was caused by rupture of an adrenocortical carcinoma. Left adrenalectomy was subsequently carried out via laparotomy.

**Conclusions:**

We experienced an unusual case of acute massive adrenal hemorrhage caused by the rupture of a non-functional adrenocortical carcinoma, which was treated successfully by ambulatory transcatheter embolization therapy and elective surgery.

## Background

Adrenocortical carcinoma is an uncommon disease, accounting for approximately 0.02% of all malignant tumors [1-3]. This tumor often exhibits endocrine activity, commonly in association with Cushing’s syndrome, because of excessive secretion of cortisol from the tumor cells. Nevertheless, non-functional tumors are also occasionally detected [1-3]. About 30% of adrenocortical carcinomas are diagnosed incidentally during imaging procedures for unrelated medical issues [4]. However, the asymptomatic nature of the tumor means that diagnosis is often delayed, and such lesions are therefore usually found only after the disease has progressed to an advanced stage, resulting in a poor outcome. Acute adrenal hemorrhage mostly occurs within adrenal tumors and the adrenal glands, often resulting in the formation of massive hematomas extending into the retroperitoneum, in association with urgent symptoms such as lower back pain, acute adrenal crisis, circulatory failure, and shock [5,6]. Anticoagulant therapy is a potential predisposing factor, but was not relevant to the present case [7]. In addition, we can leave when both adrenal hemorrhage compares it if unilateral, and adrenal insufficiency is easy to be caused [5,6].

We report an unusual case of a spontaneously ruptured, non-functional adrenocortical carcinoma that initially presented as a critical massive retroperitoneal hemorrhage, and which was successfully treated by a combination of emergency interventional radiology and elective surgery.

## Case presentation

**Figure 1:**
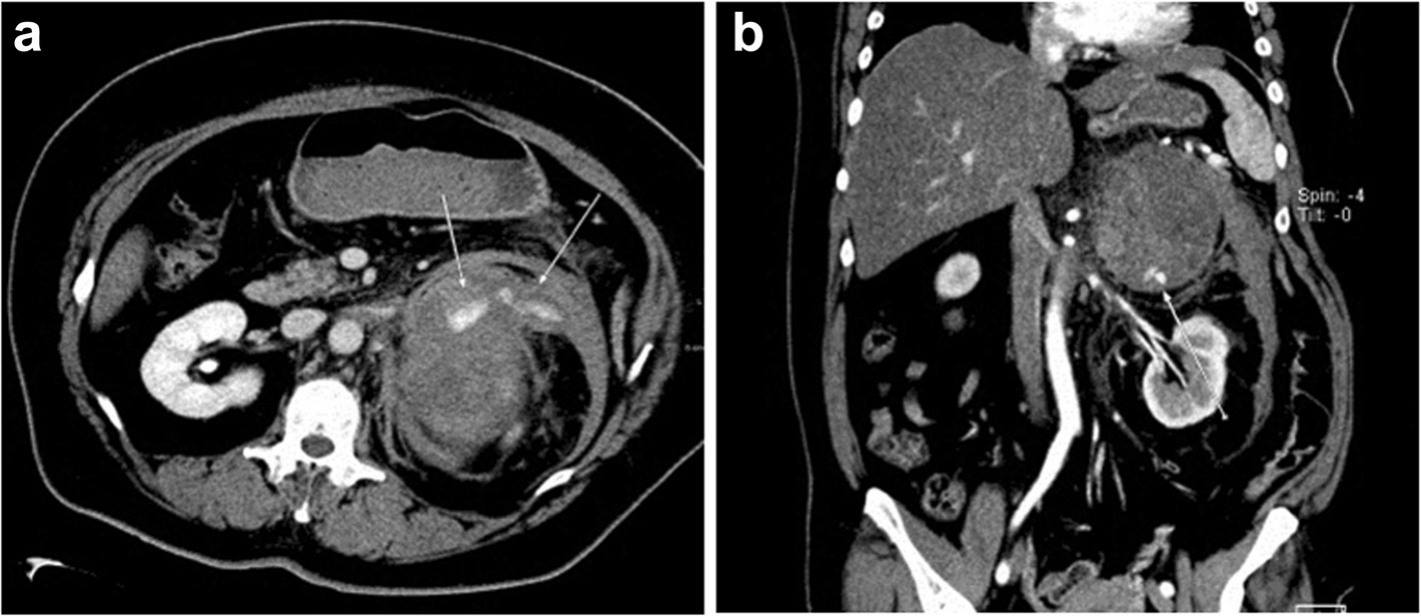
Contrast-enhanced CT findings. **a**. Emergency contrast-enhanced CT of the abdomen revealed a tumor-like soft mass accompanied by massive bleeding in the retroperitoneum (Transverse plane). **b**. Coronal plane.

**Figure 2:**
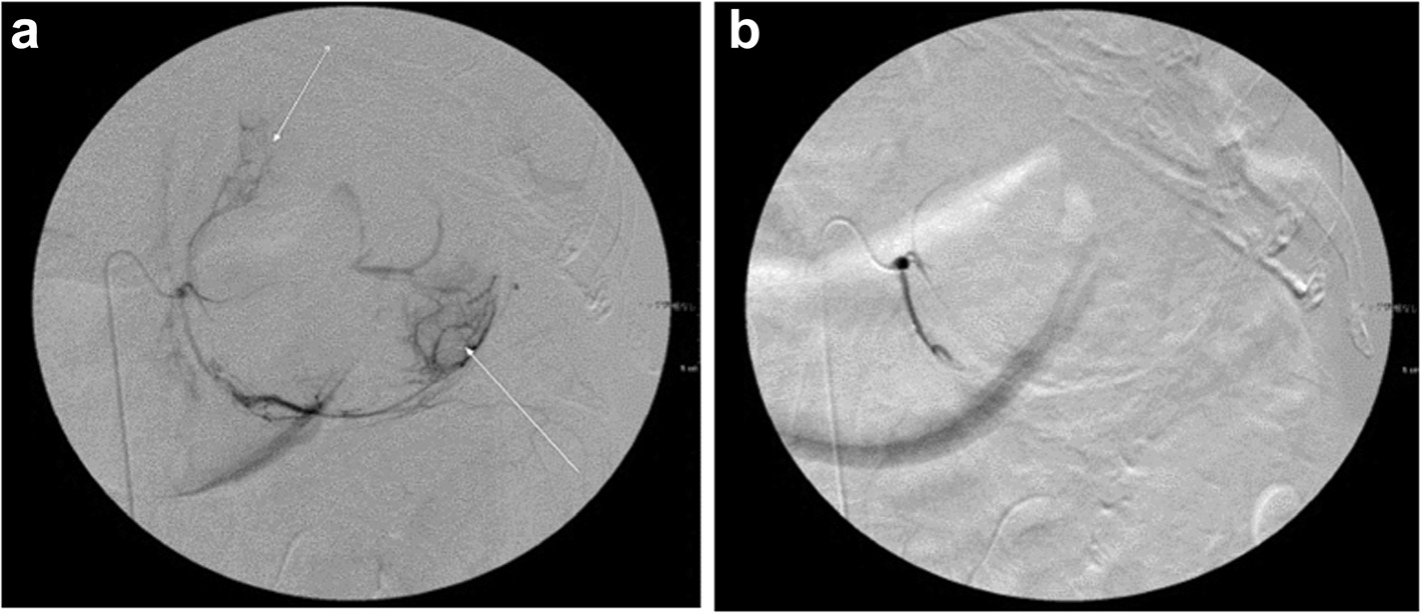
Transcatheter embolization therapy. **a**. The left middle adrenal artery was carried out under a diagnosis of acute adrenal hemorrhage (Before arterial embolization). **b**. After embolization.

**Figure 3:**
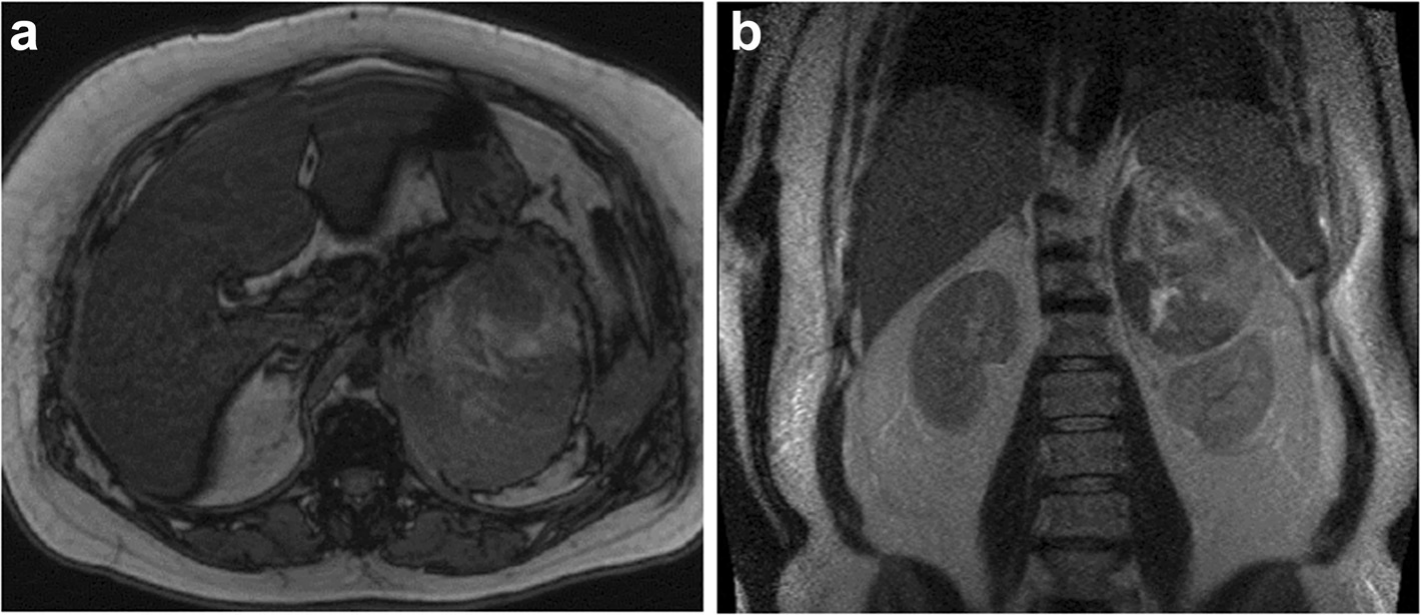
MRI images findings. **a**. A tumor exhibiting non-uniform high to low signals on both T1 and T2 MRI images was observed in the left adrenal gland (Transverse plane). **b**. Coronal plane.

**Figure 4:**
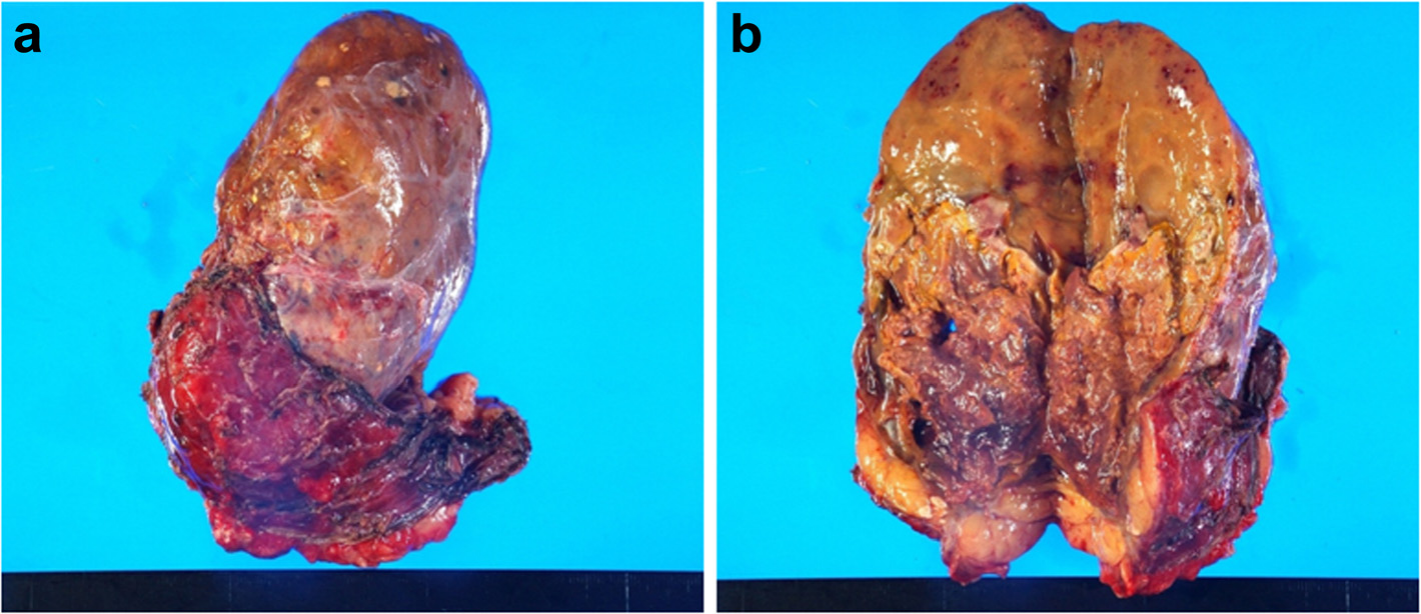
Resected specimen. **a**. The tumor measured in 13.5 × 8.6 × 4.2 cm and weighed 380 g. **b**. The cut surface was yellow and solid, and areas of bleeding accompanied by necrosis.

**Figure 5:**
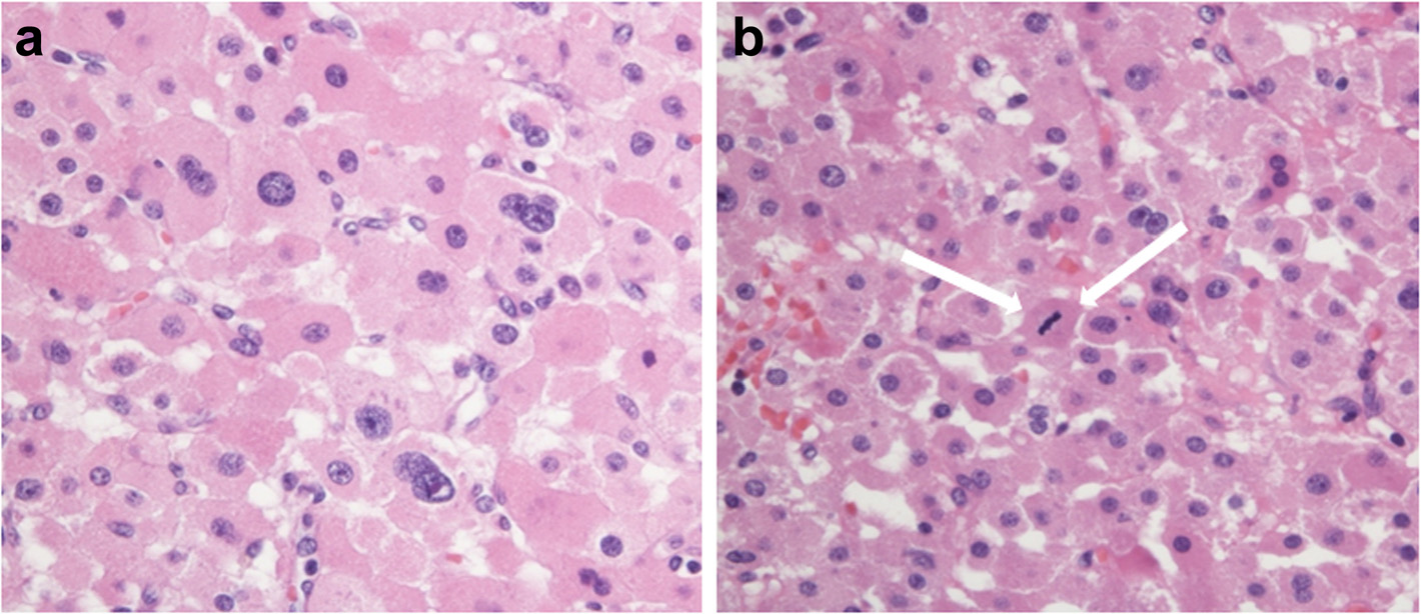
Histopathological findings. **a**. Histopathological findings. of solidly proliferating tumor cells showing an atypical morphology with an eosinophilic or clear cytoplasm and large nucleus containing a distinct nucleolus **(**Nuclear atypia: Fuhrman criteria Grade III). **b**. Arrow: atypical mitotic figures. (Hematoxylin and eosin staining × 400).

A 47-year-old woman was transported to our hospital because of the sudden onset of severe pain in her left lower back. She was alert and conscious, with a blood pressure of 165/95 mmHg, pulse of 77 beats/minute, and oxygen saturation of 95%. She appeared to be in distress and complained of sharp spontaneous pain in her left lower back. She was 162.0 cm tall and weighed 84.5 kg. There were no physical features suggestive of Cushing’s syndrome. The patient had been healthy with no particular past medical history or familial disease. Her white blood cell count was 11,800/μL and her hemoglobin level was 8.8 g/dL. Other than these findings, biochemical tests and urinalysis on admission demonstrated no particular abnormalities (Table 1). Emergency contrast-enhanced computed tomography (CT) of the abdomen revealed a tumor-like soft mass accompanied by massive bleeding in the retroperitoneum on the upper pole of the left kidney, in addition to vascular leakage from the middle suprarenal artery (Figure 1a, b). Immediately after obtaining the results of the CT scan, transcatheter embolization therapy of the left middle adrenal artery was performed based on a diagnosis of acute adrenal hemorrhage, possibly from a ruptured adrenal tumor (Figure 2a, b). The patient’s condition subsequently stabilized and a detailed investigation of the cause of the adrenal hemorrhage was initiated. Magnetic resonance imaging (MRI) revealed a hematoma expanded within the Gerota’s fascia, and a round tumor was detected in addition to the hematoma, with a rich vasculature and possible necrotic area (Figure 3a, b). No liver nodules or swelling of the retroperitoneal lymph nodes suggestive of metastasis were observed. Endocrine function of the adrenal gland appeared normal. The above findings indicated a ruptured non-functional adrenocortical tumor. Imaging confirmed the absence of metastatic lesions, and the tumor was removed by elective laparotomy surgery 2 months later. Regarding the surgical findings, no hemorrhage or abnormal adhesion was observed in the abdominal cavity. A soft elastic tumor measuring 10 cm was palpable in the retroperitoneum at the inferior border of the pancreatic body. Although it adhered closely to the surrounding retroperitoneal fatty tissue, the tumor was removed *en bloc* by partially excising Gerota’s fascia, without leaving any remnants. No invasion into the surrounding organs, lymph node metastasis, or liver metastasis was observed. The operative time was 229 min, and the surgical blood loss was approximately 500 mL. The patient recovered uneventfully. The tumor measured 13.5 × 8.6 × 4.2 cm and weighed 380 g. The cut surface indicated a yellowish solid tumor, with areas of bleeding accompanied by necrosis in a branched pattern (Figure 4a, b). The normal adrenal gland could not be identified visually. Histopathological examination revealed proliferated tumor cells with an atypical morphology, with an eosinophilic or clear cytoplasm and large nucleus containing a distinct nucleolus (Figure 5a, b). Nuclear atypia and abundant nuclear mitotic images were observed (≥11 per 50 high-power fields (HPF)). A wide focus of necrosis was also noted, although there was no apparent invasion outside the capsule. The final diagnosis was a ruptured, non-functioning left adrenocortical carcinoma, pT2N0M0, stage II. Mitotane administration for 2 years was planned as adjuvant therapy. The patient was alive at 6 months after surgery, with no serious adverse events or relapse.

**Table 1:** **Laboratory data on admission**

**【Blood count】**		**【Tumor marker】**		
WBC	11,800/μl	CEA	1.6 ng/ml	
RBC	303 × 10^4^ /μl	CA19-9	18 U/ml	
Hb	8.8 g/dl			
Hct	26.6%			
PLT	21.8 × 10^4^ /μl			
**【Blood chemistry】**		**【Endocrinology】**		(normal range)
TP	7.1 g/dl	Vanillymandelic acid	8.0 ng/ml	(3.3 ~ 8.6)
Alb	4.1 g/dl	Adrenaline	<0.01 ng/ml	(0.00 ~ 0.17)
AST	13 IU/l	Nor-Adrenaline	0.42 ng/ml	(0.15 ~ 0.57)
ALT	13 IU/l	Dopamine	<0.02 ng/ml	(0.00 ~ 0.03)
ALP	262 IU/l	Cortisol	9.2 μg/dl	(4.0 ~ 19.3)
LDH	137 IU/l	ACTH	46.9 pg/ml	(7.2 ~ 63.3)
T-Bil	0.5 mg/dl	DHEA-S	225 μg/dl	(33 ~ 262)
BUN	9 mg/dl			
		**【Urinary Cathecolamine】**		(normal range)
Cre	0.88 mg/dl	Vanillymandelic acid	3.5 mg/day	(1.4 ~ 4.9)
Na	142 mEq/l	Adrenaline	12.1 μg/day	(1.0 ~ 23.0)
K 4.	0 mEq/l	Nor-Adrenaline	94.2 μg/day	(29 ~ 120)
Cl	106 mEq/l	Dopamine	260 μg/day	(100 ~ 1000)
Ca	8.9 mEq/l	Metanephrine	0.11 mg/day	(0.05 ~ 0.20)
P	3.1 mEq/l	Normetanephrine	0.19 mg/day	(0.1 ~ 0.28)

## Discussion

Adrenocortical carcinoma is an uncommon disease found in 0.5–2 per million people, accounting for only 0.02% of all malignant tumors [1-3]. Approximately 40% of adrenocortical carcinomas have been reported to be non-functional [1-3]. In contrast, Cushing’s syndrome is often present in cases of functioning tumors [1], with dehydroepiandrosterone sulfate as a tumor marker. The prognosis of patients with adrenocortical carcinoma is poor, with a 5-year survival rate of 16–35% [8], largely attributable to the fact that approximately 80% of cases are discovered at an advanced stage [9]. In the present case, however, although the tumor was large, no extracapsular invasion or metastasis had occurred and complete resection was accomplished successfully.

CT and MRI are useful modalities for diagnosing adrenocortical carcinoma [10-12]. Tumors ≥5 cm are highly likely to be malignant, and non-uniform imaging effects/necrosis in the center accompanied by calcification have been reported to be a common finding of malignant tumors [10]. Typical adrenocortical cancer presents with low signals on T1 intensity MRI images and non-uniform high signals on T2 intensity images [11,12]. The diagnosis of non-functional adrenocortical carcinomas using adrenal cortex scintigraphy is generally regarded as difficult. However, Weiss criteria are often used to differentiate between malignant adrenocortical tumors and benign tumors pathologically [13,14]. Nine criteria are assessed, including a high nuclear grade, >5 mitoses per 50 HPF, atypical mitotic figures, <25% clear cells, a diffuse architecture, necrosis, venous invasion, sinusoidal invasion, and capsular invasion. The diagnosis in the present case was made based on four criteria: nuclear grade, mitotic index, diffuse architecture, and necrosis.

Surgical resection is the treatment of choice in patients with adrenocortical carcinoma, and survival can be prolonged significantly among patients able to undergo radical excision [1,14]. The steroidogenesis repressor mitotane is often used in cases of advanced disease, or as postoperative adjuvant therapy. Previous reports also found that the administration of postoperative adjuvant therapy following radical surgery prolonged relapse-free survival [15]. However, no universal consensus has been reached regarding the efficacy of mitotane as adjuvant therapy [4,16,17].

Adrenal hemorrhage is an uncommon entity. Although trauma is the most common cause, non-traumatic etiologies have also been reported. Large and progressively growing tumorous lesions, such as adrenocortical carcinomas, pheochromocytomas, metastatic tumors, and myelolipomas, may carry a risk of tearing or rupture, either alone or in combination with hemorrhagic diathesis, stress, or other idiopathic causes [4,16,17]. Massive adrenal hemorrhage remains rare. With respect to its treatment, follow-up is therefore often sufficient if the patient’s systemic condition is stable and findings for tumorous lesions are negative [17]. However, if the patient’s systemic condition is unstable or bleeding from a tumorous lesion is suspected, as in the current case, adrenalectomy, including the hematoma, is required [17,18]. The effective application of transcatheter embolization therapy has been reported, including in the present case, suggesting its potential value in patients with an unstable systemic condition [19-21].

We experienced an unusual case of acute massive adrenal hemorrhage caused by the rupture of a non-functional adrenocortical carcinoma, which was treated successfully with ambulatory transcatheter embolization therapy and elective surgery.

## Conclusions

We experienced an unusual case of acute massive adrenal hemorrhage caused by the rupture of a non-functional adrenocortical carcinoma, which was treated successfully by ambulatory transcatheter embolization therapy and elective surgery.

## Consent

Written informed consent was obtained from the patient for publication of this case report, including the associated images.

## Abbreviations

CT: computed tomography
MRI: magnetic resonance imaging
